# A scoping review: The utility of participatory research approaches in psychology

**DOI:** 10.1002/jcop.22231

**Published:** 2019-08-23

**Authors:** Leah Levac, Scott Ronis, Yuriko Cowper‐Smith, Oriana Vaccarino

**Affiliations:** ^1^ Department of Political Science University of Guelph Guelph ON Canada; ^2^ Department of Psychology University of New Brunswick Fredericton NB Canada; ^3^ Political Science and International Development University of Guelph Guelph ON Canada; ^4^ Department of Psychology University of Guelph Guelph ON Canada

**Keywords:** forensic psychology, HIV/AIDS, Indigenous Peoples, industrial‐organizational psychology, older adults, participatory research, research exemplars, youth

## Abstract

Consistent with community psychology's focus on addressing societal problems by accurately and comprehensively capturing individuals’ relationships in broader contexts, participatory research approaches aim to incorporate individuals’ voices and knowledge into understanding, and responding to challenges and opportunities facing them and their communities. Although investigators in psychology have engaged in participatory research, overall, these approaches have been underutilized. The purpose of this review was to examine areas of research focus that have included participatory research methods and, in turn, highlight the strengths and ways that such methods could be better used by researchers. Nearly 750 articles about research with Indigenous Peoples, children/adolescents, forensic populations, people with HIV/AIDS, older adults, and in the area of industrial‐organizational psychology were coded for their use of participatory research principles across all research stages (i.e., research design, participant recruitment and data collection, analysis and interpretation of results, and dissemination). Although we found few examples of studies that were fully committed to participatory approaches to research, and notable challenges with applying and reporting on this type of work, many investigators have developed creative ways to engage respectfully and reciprocally with participants. Based on our findings, recommendations and suggestions for researchers are discussed.

## 1 INTRODUCTION

Across the social sciences, growing attention is being paid to the role of Participatory Research (PR) approaches and research informed by PR principles^1^ in advancing both knowledge and practice. Discussed further below, PR principles include reciprocity of benefit and capacity building (Belone et al., 2016), research partner and participant involvement across research stages (Cook, 2008; Viswanathan et al., 2004), and acknowledgment of power differentials between academic researchers and researcher participants (Belone et al., 2016). In community psychology (CP), where advances in theory, research, and interventions are informed by practices that privilege people's lived experiences, there is a long history of research informed by PR principles (Gregory, 1999; Yen, 2007). However, the knowledge and action benefits that result from PR are still sidelined in many areas of psychology research. Moreover, there are no studies^2^ that take stock of the breadth and quality of the application of PR principles.

To address this gap, we answer two questions: (a) How are PR principles being used in six key areas of research focus, and (b) across these areas, which studies can be considered exemplars? In answering these questions, we aim to provide a broad picture of the use of PR approaches in psychology and to offer psychologists a framework for considering ways in which their own research may be strengthened through the introduction of PR principles. Furthermore, we hope this review encourages the application of PR principles across more stages of the research process and across a broader swath of psychology research.

To address our research questions, we began by undertaking an extensive scoping review, the results of which highlighted *where PR is being used* in psychology research*.* We identified six areas of research focus where we expected PR was being used and coded almost 750 articles across these areas. Next, we examined *how PR is being used in these areas of research focus,* such as in building long‐term partnerships to address intractable community challenges, developing and evaluating interventions, or better understanding the experiences of historically marginalized groups such as sex workers, youth experiencing homelessness, and Indigenous Peoples. Finally, drawing on social science literature that describes promising practices in the application of PR principles, we present examples of psychology research that highlight PR principles.

### 1.1 Background and theoretical framing: Participatory research in psychology

The roots of PR can be traced to a number of fields, including action research, adult education, critical sociology, and international development (Brown & Tandon, 1983; Kagan, 2012; Khanlou & Peter, 2005). PR originated in an atmosphere of increased concern for respect of individuals, beneficence, and justice for vulnerable populations, following several examples of extremely unethical or abusive practices in social science research, such as the Tuskegee Syphilis Study or the experimentation conducted on people in Nazi concentration camps (Blumenthal, Hopkins, & Yancey, 2013; Khan et al., 2005; Manton et al., 2014). PR offers a way of collaborating with community members in the design and conduct of studies, as well as in the dissemination of research findings (Cargo & Mercer, 2008; Creswell, Hanson, Clark Plano, & Morales, 2007; Morris, 2002; Wint, 2011). Instead of being understood as research subjects, participants are involved as co‐researchers and recognized as having intimate experiential knowledge and robust knowledge systems. Their expertise positions them as important actors in developing and undertaking multiple aspects of projects (Morris, 2002).

### 1.2 Community psychology: A comfortable but narrow home for participatory research

The field of Community Psychology (CP) began with an aim of confronting and challenging unjust social institutions in response to community‐based protest (Gregory, 1999; Yen, 2007). For example, in response to social issues such as poverty, racism and sexism, the National Institute of Mental Health sponsored the Swampscott Conference in 1965 (Yen, 2007). The Conference focused on deinstitutionalization and community mental health, and created a platform for discussing the social origins of mental health and illness, and social interventions (Walsh, 1987). This conference formed the basis of the CP movement and reconceptualized the role of psychologists (Gregory, 1999). Soon after, in 1967, the American Psychological Association (APA) established a division for CP (Newbrough, 1997).

In the following decades, the field of CP grew and broadened in scope to welcome a range of ideas and methods to the discipline (Sigogo et al., 2004; Yen, 2007). This included PR methods, which were aligned with CP's aim to give voice and power to affected groups (Arcidiacono & Procentese, 2010). Overall, the role of PR in CP has been to advance community interests and social justice (Kagan, 2012). In many countries, including the United States, the development of CP was accompanied by the rise of a focus on civil and human rights (Gregory, 1999; Walsh, 1987). In the 1970s, in countries in both the global north and global south, work in CP grew in reaction to systemic political repression and exclusion from access to resources and power (Wingenfeld & Newbrough, 2000; Yen, 2007; Zuniza, 1975). These elements and origins of CP fit with the social justice orientation of PR.

### 1.3 Participatory research in broader psychological research

Although there is no consensus regarding the standards of PR approaches across the social sciences or in psychology in particular (Creswell et al., 2007; White, Suchowierska, & Campbell, 2004), seven principles outlined by Hall over 40 years ago – including providing direct benefits to the community and building ongoing relationships between researchers and community members – continue to be considered foundational (Creswell et al., 2007; White et al., 2004). Taken in combination with others’ frameworks of participatory approaches (e.g., Blumenthal, 2011; Corbett, Francis, & Chapman, 2007; Ochocka & Janzen, 2014), several principles are salient, including: community member involvement across research stages, knowledge sharing – and thus respect – between involved community members and researchers, sound ethical principles, and concrete steps toward positive social change. Further, scholars have focused on the challenges of applying, and need for better understanding, the complexity of these principles. For example, Campbell (2016) calls for advancing an ethical framework for undertaking CP research, and Lykes (2017) reminds readers of the challenges of managing power differentials between academic researchers and community members and recommends “re‐politicizing” PR.

Despite many benefits, there are several critiques or challenges to PR approaches. A common concern relates to the scientific stringency of PR as understood by positivist approaches. Indeed, many researchers are trained to maintain “objectivity”. Positivist researchers are generally suspicious of whether PR adheres sufficiently to a scientific outlook and methodology. Participatory approaches are also critiqued for potentially providing only a partial or tokenized voice to participants and for struggling to meaningfully address power relations that remain embedded in the “politics of knowledge production” (Guishard, 2009). Given that PR includes participants in the same arena as the researcher and that everyone may have different interests in the research, concerns about competing influences on research outcomes are also raised (Mackenzie, Tan, Hoverman, & Baldwin, 2012). Ultimately, PR adopts a critical or post‐positivist position, which calls for the investigator to become keenly aware of power dynamics that imbue and sustain society (and research processes) as we know them. These underlying ideological positions are more in line with the commitments of PR, which, as noted above, include advancing social justice and engaging meaningfully with participants, particularly those who have – and continue – to experience marginalization and colonization.

Within these critical research orientations, participatory researchers undertake extensive efforts to ensure research rigor. For example, university‐based researchers learn from community members to understand appropriate community protocols, and in turn train community participants on relevant research protocols (e.g., Levac, 2013). They also follow guidance from scholars such as Wicks and Reason (2009), who highlight the importance of developing safe and open communicative spaces with research participants, including through the stages of inclusion, control, and intimacy. Further, tools such as memoranda of understanding can be used to guard against the concern of competing interests (Mackenzie et al., 2012), and ethical principles such as Ownership, Control, Access, and Possession (OCAP, 2018), which contribute to governing research with Indigenous Peoples, are increasingly common. Further, PR processes tend to follow the same general process of scientific inquiry as does positivist research, including problem definition/question formulation, information gathering, and analysis (Ochocka & Janzen, 2014), and some researchers argue that participatory approaches actually improve research rigor by ensuring results are valid for the community (Warr et al., 2018).

Some investigators argue that PR should include community members’ participation and input in every research stage (Morris, 2002; Viswanathan et al., 2004; Wint, 2011), but very few studies in the psychological literature meet this standard (Catalani & Minkler, 2010; Morris, 2002). Viswanathan and colleagues (2004) found only three studies out of 60 in which community participants were involved from beginning to end. In their review of 46 studies, Westfall, Van Vorst, Main, and Herbert (2006) noted that none fulfilled the criteria of involving community members at every stage. We agree that PR – at its best – ensures mutuality and collaboration across all research stages. However, because this is rare, while we pursue the longer‐term goal of fully implemented PR, we suggest that it is also important to find ways to share power between persons, groups, and institutions, and maintain ongoing, collaborative communication, even when full collaboration at all research stages is elusive.^3^

## 2 METHODS

The research presented in this article charts a pathway from understanding principles of PR to examining their application across a broad swath of psychology research. We undertook this study in three phases.

### 2.1 Phase 1. Scoping review

In October 2016, the research team conducted an initial review of four major psychological indices: PsychINFO, ERIC, MedLine, and Web of Science. The search was limited to empirical journal articles to target research studies and limit other kinds of publications. Our inclusion and exclusion criteria were initially very broad, beginning with the collection of articles that contained the words “participatory” and “research”. This initial search yielded over 3,500 articles across 10 broad research fields including: Clinical psychology, community psychology, cultural/cross‐cultural psychology, developmental psychology, educational psychology, environmental psychology, forensic psychology, health and medicine, industrial/organizational psychology, and psychology research with marginalized populations. To narrow our focus, we undertook several steps. First, we organized the studies uncovered into the categories listed above. Then, by scanning the abstracts, we subdivided articles within each field into areas of research focus. For instance, in the “community psychology” field, articles were grouped into three subgroups or research areas, including: Advocacy, community planning/development, and social work and services. Next, we inductively selected six research areas of focus from across the identified fields, including: Child and adolescent research, research with Indigenous Peoples,^4^ research with older adults, industrial/organizational research, research with people living with HIV/AIDS, and forensic research. These six research areas of focus were included because: They were among the areas with the most examples; they covered a broad spectrum of methods (e.g., quantitative and qualitative); there was minimal overlap between them; and they spanned several unique contexts. We also examined other research areas of focus but determined that there was sufficient overlap with the six selected foci in terms of methodological approaches and/or general content (e.g., marginalization and health) to ensure that our review would be generalizable to other areas while maintaining concision.

Across these foci, our search yielded 1,475 articles, which were imported and stored in RefWorks. Despite the large number of articles uncovered, we decided to retain our focus across these areas for two reasons. First, so doing offered the possibility of identifying common and unique PR approaches which could in turn offer expanded methodological and principled guidance to university‐based researchers. Second, these foci reflect areas where university‐based researchers must overcome long and problematic histories of extractive and harmful research practices, and thus where PR approaches are especially critical.

The abstract and method section of each article was reviewed to determine both its use of PR approaches and its status as a psychology study. Included articles were those that referenced participatory research in the abstract or that described some dimension of participant collaboration in the method section. Excluded articles were those that did not reference participatory research in the abstract or in the method section. Editorials, commentaries, and reviews were also excluded, though we retained review articles for our own reference, and to contribute to the framing of this article. After we established the inclusion and exclusion criteria, we divided the original 1,475 articles into four approximately equal chunks. To achieve inter‐rater reliability, four team members tested the inclusion/exclusion logic by reviewing the abstract and method section of the same 10 articles. This exercise allowed the researchers to create a common understanding of how to apply the inclusion/exclusion criteria. In cases of disagreement, the two lead authors made the final decision. In cases of uncertainty, all team members erred on the side of inclusion. Ultimately, this process yielded 749 articles for inclusion.

### 2.2 Phase 2. Defining exemplars

As noted above, one of our goals was to identify studies that could be used as promising examples of participatory approaches being used by investigators in various content areas of psychological research. Drawing on recent research about criteria for assessing high quality PR (Belone et al., 2016; Lucero et al., 2016), along with an extensive comprehensive review of community‐based PR (Viswanathan et al., 2004), we used three criteria to help identify exemplary studies:

(1) Evidence of rigorous study design, including: Identification and articulation of methods appropriate to the question; demonstration of a blend of flexibility and consistency to respect participants’ interests; and recognition of, and response to, potential challenges of PR (Viswanathan et al., 2004);
(2) evidence of community/participant involvement in all research stages; for example, community partners having influence in research design, problem definition, and research decision‐making, and being acknowledged as experts (Cook, 2008; Viswanathan et al., 2004); and
(3) evidence of partnerships that encourage trust, promote reciprocal capacity building, and acknowledge power dynamics (Belone et al., 2016).

To look for evidence of these criteria, we read the method section of each article thoroughly to search for evidence that the study had been designed with careful attention to the participants’ preferences. We also established that both the relationship between the question and the methods, and the context (criterion 1), were clear, such as is described by Allen, Mohatt, Beehler, and Rowe (2014a) in the “People Awakening Project.” We also looked for explicit descriptions of participant involvement at all stages (criterion 2), such as when Maglajlić and RTK PAR UNICEF BiH Team (2004) describe the role played by each team member.

### 2.3 Phase 3. Coding and identification of exemplars

The 749 included articles were imported into NVivo© and coded using a series of 10 nodes. Six nodes referred to instances of the application of PR approaches in six key stages of the research process, including: (a) design, (b) recruitment, (c) data collection, (d) data analysis/interpretation, (e) implementation (applicable only to intervention studies), and (f) dissemination (sometimes described as knowledge mobilization). An additional three nodes were used to identify exemplars, following the criteria noted above in the description of Phase 2. A study could also fail to meet any of our criteria for being exemplary. A tenth node, “No PR in Study,” was added to tag articles that did not clearly articulate the application of participatory approaches – exemplary or otherwise – in any of the six stages of research, despite having initially been tagged for inclusion.

## 3 RESULTS

### 3.1 Intersection of research areas of focus and PR approaches

Table 1 presents an overview of the number of studies uncovered in each of the six research areas of focus, including the number of studies retained in each category throughout the review process. Since we do not know the total number of articles published in each content area over the same time period, we cannot speak to the proportionality of the use of participatory principles in any given area. However, a query of PsycINFO seeking all peer‐reviewed psychology articles from 2012 to 2017 generated 915,375 results, suggesting that by any measure, the number of articles tagged using “participatory” and “research” is miniscule. It is nevertheless notable that the 749 articles included in our study are heavily concentrated (67%) in two research areas: Research with children and adolescents, and research with Indigenous Peoples. In the case of the former, the slow realization that inclusion of children's and youth's perspectives is crucial for understanding psychosocial phenomena in these populations may help explain the volume of investigations using participatory principles in at least some stage of research. At the same time, gaps remain and meaningful engagement directly with children and youth by researchers still appears to be somewhat elusive. In the case of the latter, this is not surprising given the amount of work Indigenous researchers and community members have taken on to reveal and reject the persistence of colonization, and related exploitative research *on* Indigenous People across North America. Indeed, “among Indigenous People in Canada, this unidirectional gaze is no longer tolerated” (Ball & Janyst, 2008, p. 38). Further, the associated responses by Indigenous Nations, governments, and organizations to abusive research practices has increasingly been to insist on collaborative research protocols such as the OCAP® Principles (2018) and those set out by Inuit Tapiriit Kanatami (n.d.).

**Table 1:** Results of scoping review

	# of Articles included on search of “Participatory” and “Research”	# of Articles included on abstract (cross sub‐field duplicates)	# of Articles included on review of methods
Indigenous Peoples and communities	238	174	160
Child/adolescent development	715	354	345
Forensic (incarcerated people, sex workers, etc.)	58	37	29
HIV/AIDS	190	85	60
Older adults	128	88	72
Industrial/organizational	146	89	83
		Total # of articles in study	749

Table 2 shows how often PR principles were incorporated across the six main stages of research in each of the six research areas of focus we explored. How these principles were applied is discussed in more detail below. Overall, we find that participatory principles are most likely to guide research design, and least likely to inform participant recruitment. Just over half (56%) of the studies we reviewed discussed the use of PR principles in designing the project. Studies categorized as “forensic” and studies with Indigenous Peoples were most likely to be designed collaboratively (66% and 64%, respectively), whereas collaborative data collection was most common in studies with older adults (63%) or with people living with HIV/AIDS (72%). Half of the research we reviewed applied participatory principles to data collection. Only 17% of all studies reviewed discussed participatory approaches to participant recruitment, which is somewhat surprising given that established recruitment strategies, such as snowball sampling, rely on participants inviting other participants. It is also the case that there is relatively little evidence of collaborative research dissemination, raising questions about how findings might be serving the needs and experiences of research participants and their peers.

**Table 2:** Participatory approaches in six research areas in psychology

	Total # of articles included	# of Articles with PR in design (%)	# of Articles with PR in recruitment (%)	# of Articles with PR in data collection (%)	# of Articles with PR in analysis/interpretation (%)	# of Articles with PR in implementation (%)	# of Articles with PR in dissemination (%)
Indigenous Peoples and communities	160	103 (64)	13 (8)	61 (38)	38 (24)	13 (8)	24 (15)
Child/adolescent development	345	203 (59)	80 (23)	192 (56)	143 (41)	99 (29)	97 (28)
Forensic (offenders, sex workers, etc.)	29	19 (66)	1 (3)	9 (31)	4 (14)	2 (7)	0 (0)
HIV/AIDS	60	23 (38)	9 (15)	43 (72)	22 (37)	11 (18)	2 (3)
Older adults	72	29 (40)	15 (21)	45 (63)	26 (36)	6 (8)	6 (8)
Industrial/ organizational	83	46 (55)	11 (13)	25 (30)	21 (25)	18 (22)	14 (17)
Total	749	423 (56)	129 (17)	375 (50)	254 (34)	149 (20)	143 (19)

*Note*: Column totals do not add to total number of articles included because articles may be tagged multiple times.

### 3.2 Use and utility of participatory research principles

In response to our first research question *– How are PR principles being used in these research areas of focus?* – we found examples of participatory principles being incorporated in all stages of research. The exception was forensic psychology, where we did not identify any examples of participatory principles being used in dissemination, and only one example of participatory principles being used in participant recruitment. While the application of PR principles remains relatively uncommon in psychology research, there are sufficient examples from which we can learn.^5^

#### 3.2.1 PR design

Research involving participants in the design phase often took the form of developing collaborative teams that included both academic (and often lead) researchers and local representatives from the community or population of focus. These teams typically relied on regular contact (i.e., meetings, conferences, and focus groups) to discuss and resolve challenges in research design and at other stages of the projects. The formation of advisory groups, comprised of both academic and community participants, was common in research with Indigenous Peoples (e.g., McWhirter, Mununggirritj, Marika, Dickinson, & Condon, 2012; Novins et al., 2012; Palacios & Kennedy, 2010; Richards & Mousseau, 2012; Smith, Christopher, & McCormick, 2004), HIV/AIDS‐focused studies (e.g., Abelsohn et al., 2015; Andrasik et al., 2012; Baptiste et al., 2006; Burkhalter et al., 2013; DiStefano et al., 2012; Nyamathi et al., 2004; Vallely et al., 2009), and research with older adults (e.g., Guo & Phillips, 2006; Klemm, Rempusheski, & Teixeira, 2013; Ottmann, Laragy, Allen, & Feldman, 2011; Perry & Ziemba, 2014; Scharlach & Sanchez, 2011; West & Graham, 1999; Wethington et al., 2007). These efforts were sometimes described as attempts to address inherent power imbalances between university researchers and participants (e.g., Tobias, Richmond & Luginaah, 2013). The creation of advisory groups was also present in child and adolescent research (e.g., Amsden & VanWynsberghe, 2005; Breland‐Noble, Bell, Burriss & Poole, 2012; Dworski‐Riggs & Langhout, 2010; Pavkov, Priest, & Fox, 2012; Sangalang, Chen, Kulis, & Yabiku, 2015; Tuck, 2012), and industrial/organizational research (e.g., Hammel, Finlayson, & Lastowski, 2003; Janzen, Nelson, Hausfather, & Ochocka, 2007; Scholl, 2004). A few forensic psychology projects described being guided by community‐university research teams (e.g., Brown, Varcoe, & Calam, 2011; Fields, González, Hentz, Rhee, & White, 2008) or community advisory boards (e.g., Hatton & Fisher, 2011; Lorway et al., 2014), or including research participants in the study design in other ways (e.g., Livingston et al., 2014; Varcoe, 2006; Viswanathan et al., 2004). As well, some industrial/organizational studies described research questions as emerging from the participants’ communities (e.g., Beardwood, Kirsh, & Clark, 2005; Beck, Lengnick‐Hall, & Lengnick‐Hall, 2008; Bridges & Meyer, 2007; Brow et al., 1983; Burns, Hyde, Killett, Poland, & Gray, 2014; Caister, Green, & Worth, 2012; Gouin, Cocq, & McGavin, 2011), as did some projects with children and youth (Christiansen, 2010; Dyrness, 2008; Guishard et al., 2005; Johnson‐Burel, Drame, & Frattura, 2014; Sangalang et al., 2015; Schinke et al., 2010), and with Indigenous Peoples (e.g., Brown et al., 2011; Brussoni, Olsen, & Joshi, 2012).

As noted above, over half of the studies with Indigenous Peoples reported using collaborative design processes (e.g., Allen et al., 2014a; Bainbridge et al., 2013; Brown et al., 2011; Smylie, Kaplan‐Myrth, & McShane, 2009). Besides forming advisory groups, collaborative study design with Indigenous Peoples or Nations often involved meetings with Elders (e.g., Isaak et al., 2010; Johnson, Bartgis, Worley, Hellman, & Burkhart, 2010; Jumper‐Reeves, Dustman, Harthun, Kulis, & Brown, 2014; Potv et al., 2003), establishing open and iterative processes of discussions among researchers and community participants (e.g., formal and informal Nation leadership, and general community members), and working with language interpreters or cultural advisors to identify key areas of research interest and best practices for collecting and using information. Collaborative design processes were also identified as being useful for selecting appropriate underpinning theoretical research frames (Allen et al., 2012; Allen & Mohatt, 2014). Another important consideration at the design stage related to determinations about data use and ownership (e.g., Smith, Varcoe, & Edwards, 2005; Thomas et al., 2009; Thomas, Rosa, Forcehimes, & Donovan, 2011).

In keeping with PR principles, study designs were also often described as being iterative and as occurring in phases. The goal of this approach was to achieve consensus and accuracy in the research, also sometimes termed transactional validity (Schinke et al., 2010). As an example, Annear, Keeling, and Wilkinson (2014), in their study of the role of older adults in disaster recovery, described a phased approach to their research “to facilitate cycles of planning, action, and reﬂection; to accommodate an evolving process that employed mixed methods; and to facilitate the translation of research data into recommendations for action” (p. 44). In a study with young mothers, McKay et al. (2011) described how the participating young mothers drove decisions around budget planning for the project, spending of resources, gathering key information, and mobilizing themselves for social action. Authors in other content areas also discussed using iterative models (e.g., Allen et al., 2014a; Brown et al., 2011; Ottman et al., 2011; Remien et al., 2013; Tolson, Irene, Booth, Kelly, & James, 2006).

According to authors from across the research areas of focus we examined, including participants in research design offers many benefits. For example, in their description of the community‐based development of a survey about the connections between HIV/AIDS and broader health issues, Abelsohn et al. (2015) explain that participants’ “feedback transformed the language used throughout the survey to ensure that it was inclusive to the diversity…” (p. 564). Nyamathi et al. (2004) describe a similar process for modifying a semi‐structured interview guide. Participant inclusion in research design can also help modify interventions for new contexts (Remien et al., 2013), identify appropriate and culturally relevant data collection methods (e.g., Aronowitz, Todd, Agbeshie, & Rennells, 2007; Harrison & Brandling., 2009; Schinke et al., 2010) and research priorities (e.g., Baptiste et al., 2006; Wethington et al., 2007), build skill sets in youth participants (Mordock & Krasny, 2001), empower traditionally marginalized participants (Taggart, Franks, Osborne, & Collins, 2013), resist framings of older adults as helpless (Shura et al., 2011), help projects build positive reputations in the communities in which they operate (Rhodes et al., 2012), and facilitate future uptake of results (Baumann et al., 2012).

#### 3.2.2 Participatory recruitment and data collection

Limited research collaborations included community partners in participant recruitment, but many more (50% of studies reviewed) included participants in data collection. In research focused on older adults or Indigenous Peoples, participants’ involvement in recruitment was both formal (e.g., Brown et al., 2011; Mohatt et al., 2008; Rink et al., 2012) and informal (e.g., Goins et al., 2011; Mendenhall et al., 2010; Westwood, 2013). In a study focused on connecting Indigenous Youth and Elders, Wexler (2011) described an interesting recruitment process whereby “young people were first interviewed…and then were asked to identify and recruit adults and Elders in their community that they felt could talk about resilience…” (p. 252). HIV/AIDS‐focused research teams worked with staff of partner organizations to undertake recruitment (e.g., Bowden et al., 2006; Lesch, Singh, Kafaar, Swartz, & Menezes, 2013), used respondent‐driven and network‐type sampling methods (MacQueen et al., 2015; Mill et al., 2010), and undertook methods, such as peer ethnography (Hawkins, Price, & Mussá, 2009), that are inherently participatory in their approach to recruitment and data collection. Industrial/organizational research also engaged participants in recruitment, such as by involving participating workers in hosting workshops (Beardwood et al., 2005) or focus groups (Oades, Law, & Marshall, 2011) for other workers, and using participants to establish contact with other potential participants (Bridges & Meyer, 2007). Similarly, some researchers (e.g., Guishard et al., 2005; Levac, 2013; Maglajlic, 2010; Mooney‐Somers et al., 2015; Puig, Erwin, Evenson, & Beresford, 2015; Wershler & Ronis, 2015) described training youth to recruit other youth who then conducted interviews with their peers. In general, working with youth‐serving or youth‐focused community organizations was a commonly described strategy for recruiting adolescent research participants. Specific strategies discussed ranged from hiring other youth involved in the partner organization to recruit their peers (e.g., Flicker & Guta, 2008; Kadi‐Hanifi et al., 2014; Wershler & Ronis, 2015), to integrating “[the research project] into existing program planning” (Flicker et al., 2008, p. 289). As a creative approach in data collection, Tanjasiri et al. (2013) described how youth fitted with GPS devices went around their communities and inputted waypoints according to perceived pro‐ versus antitobacco marketing.

Participants’ involvement in data collection was most common in HIV/AIDS‐focused research. Of the 60 included HIV/AIDS research articles, 72% described participants’ inclusion in data collection. This included collaborating with participants to conduct interviews (e.g., Andrasik et al., 2012; Mill, 2001), focus groups (Aronowitz et al., 2007), workshops and training (e.g., Caffrey et al., 2009; Morisky et al., 2010; Wainberg et al., 2007), peer ethnography (Hawkins et al., 2009), and needs assessments (Maher, Coupland, & Musson, 2007). Several studies also discussed the use of photovoice and other visual participatory methodologies (e.g., de Lange, 2008; Hergenrather, Rhodes, & Clark, 2006; Jacobs & Harley, 2008; Mamary, McCright, & Roe, 2007; Muthukrishna & Ramsuran, 2007; Pritzker, LaChapelle, & Tatum, 2012; Schrader, Deering, Zahl, & Wallace, 2011; Teti, Pichon, Kabel, Farnan, & Binson, 2013; Vaughan, 2014). Some forensic psychology studies also employed participatory approaches to data collection. For example, Fields et al. (2008), and Torre and Fine (2005) described engaging team members who were incarcerated in developing interview questions and undertaking interviews. Livingston et al. (2014) described similar participation in data collection of people living with mental illness. Overall, only about 15% of the 72 articles about the experiences of older adults included participants directly in data collection. In these cases, participant researchers were most commonly identified as being involved in conducting interviews (e.g., Barnes, Taylor, & Ward, 2013; Doyle & Timonen, 2010; Fenge, 2010; James, Blomberg, Liljekvist, & Kihlgren, 2015; Scharlach & Sanchez, 2011; Tanner, 2012), though arts‐based approaches were also employed in some research with older adults (Baker & Wang., 2006; Novek et al., 2012).

In contrast to their relatively limited use in research with older adults, arts‐based methods were widely used as collaborative data collection methods in research with children and adolescents (e.g., Aldridge, 2007; Amsden & VanWynsberghe, 2005; Biag, 2014; Brazg, Bekemeier, Spigner, & Huebner, 2011; Cheney, 2011; Christiansen, 2010; Clark, 2011; Dean, 2007; Enright & O'Sullivan, 2013; Findholt, Michael, & Davis, 2011; Foster‐Fishman, Nowell, Deacon, Nievar, & McCann, 2005; Garner & Faucher, 2014; Hennessey et al., 2010; Langhout, Collins, & Ellison, 2014). Although photography was the most commonly described arts‐based method, participatory mapping (Clark, 2011; Lambert, Coad, Hicks, & Glacken, 2014), drawing (Gibson, Aldiss, Horstman, Kumpunen, & Richardson, 2010), arts‐informed workshops (Christiansen, 2010), scrap‐booking (Harvey, Wilkinson, Pressé, Joober, & Grizenko, 2014), role play and story‐telling (Hartley, Murira, Mwangoma, Carter, & Newton, 2009), and popular theater (Mabala & Allen, 2002) are examples of other participatory arts‐based methods discussed in the literature.

One strategy discussed as offering capacity building opportunities and promoting accurate and valid data collection, was inviting participants to attend workshops focused on developing research skills, establishing rapport, asking appropriate follow‐up questions, and validating emerging themes (e.g., Harper, Jamil, & Wilson, 2007; Levac, 2013; Puig et al., 2015; Vaughan, 2014). In a paper reflecting on the implementation of several related sports‐focused PR projects, Blodgett et al. (2011a) explained that “the conversational interviews [used in the research] served to empower the Aboriginal co‐researchers to lead the interview process from within a relevant, local worldview” (p. 268). Training and employing community members to engage in data collection was a strategy used in other research with Indigenous People as well (e.g., Daley et al., 2012; English et al., 2008; Fleischhacker et al., 2012; Flint et al., 2011; Jacklin & Kinoshameg, 2008; Makosky daley et al., 2010; Ng, 2012; Patrick & Budach, 2014; Varcoe et al., 2013; Wexler, 2011).

Discussions about participatory recruitment and data collection included specific consideration of consent processes, and questions of ethics. For example, in a study with older adults, Tanner (2012) described using a “process model of consent” (p. 297), where consent was revisited throughout the research process. James et al. (2015) discussed the ethical challenges of including “older persons with cognitive impairment, which may be considered unethical…. [to ensure ethical contact], research facilitators studied whether the older persons had any hesitation about participating and reiterated that they could end their participation at any time” (p. 342).

#### 3.2.3 Participatory data analysis/interpretation

Just over one‐third (34%) of articles reviewed across content areas included participatory approaches to data analysis and interpretation. Approaches included: Discussions among team members (e.g., Mill, 2001; Mill et al., 2010; Wainberg et al., 2007), joint coding (e.g., Andrasik et al., 2012; Burkhalter et al., 2013; Lorion, 2004; Maher et al., 2007; Matebeni, Reddy, Sandfort, & Southey‐Swartz, 2013; Mohatt et al., 2004), “talking circles” (Schinke et al., 2010), and interpreting maps (Amsden & VanWynsberghe, 2005) and images (Foster‐Fishman et al., 2005; Jacobs & Harley, 2008). As well, the participatory nature of the arts‐based photovoice method includes participant‐led analysis of the meaning of their photos (e.g., Aldridge, 2007; Garner & Faucher, 2014; Taggart et al., 2013; Vaughan, 2014).

Cotterell (2008) describes a robust process of collaborative and iterative analysis including a “series of 10 analysis sessions in which all transcripts were analyzed in turn, four theme generation sessions, one session to expand or collapse the analysis into ﬁnal themes, and, lastly, a session to integrate phase one and two themes where possible” (p. 667). Hutnik, Smith and Koch (2012) describe an interesting process of iterative story creation, and Castleden, Garvin, and Huu‐ay‐aht (2008) as well as Jacklin and Kinoshameg (2008) describe training community members to work as data analysts. Some researchers working with Indigenous Peoples (e.g., Allen et al., 2014a, 2014b; Blodgett et al., 2011a; Blodgett, Schinke, Smith, Peltier, & Pheasant, 2011b; Brown et al., 2011; Smylie et al., 2009) specifically attended to prioritizing community members' interpretations over those of academics, particularly when there were discrepancies between the two perspectives.

While data analyses can clearly be participatory, participants were more commonly asked to reflect on the data collected or on the interpretations of researchers’ analyses (and thus codes; e.g., Byers, Kulitja, Lowell, & Kruske, 2012; Cross et al., 2011). For youth in particular, with few exceptions (e.g., Dunn, Niens, & McMillan, 2014; Flicker et al., 2004; Flicker et al., 2008; Khanare, 2012; Langhout et al., 2014; Levac, 2013), reflections often came from adults (e.g., professionals working with youth), and not directly from youth (e.g., Fournier, Bridge, Pritchard Kennedy, Alibhai, & Konde‐Lule, 2014; Mooney‐Somers et al., 2015; Russell, 2004). Cross (2009) described introducing follow‐up interviews in a study “to let children's own commentary and evolving narratives serve as the primary source of the interpretive frame” (p. 338). Overall, the relative lack of youth voice in data interpretation is unfortunate given the observation by Amsden and VanWynsberghe (2005) that “what we discovered…was that despite the looseness of this [analytical] structure, there were many consistencies in terms of themes overall” (p. 364), and the proposition “that engaging youth in meaning extraction through data analysis is an essential, though often forgotten, step…” (Foster‐Fishman et al., 2010, p. 75). In other words, young people can contribute to understanding research results.

More limited forms of participant involvement in analyses of research with older adults and in research with people living with HIV/AIDS included inviting participants to critique and elaborate on findings (e.g., Annear et al., 2014; Lesch et al., 2013; MacLeod, Skinner, & Low, 2012; Mamary et al., 2007; Nomura et al., 2009; Scharlach & Sanchez, 2011; Tolson et al., 2006; Zakrajsek, Schuster, Guenther, & Lorenz, 2013). Only three forensic psychology studies we uncovered described the involvement of participating prisoners (Hatton & Fisher, 2011) and sex workers (Shannon, 2014; Viswanathan et al., 2004) in data analysis. Some research described inviting participants or community members to review reports before publication (e.g., Lantz et al., 2003; Wahab, 2004), though we suggest that this be understood as a very minimal standard of involvement.

#### 3.2.4 Participatory implementation

Participatory implementation refers to intervention studies where programs were being developed, implemented, and/or evaluated. About 20% of the studies we reviewed offered ideas about participatory approaches to implementation. These included: The creation of “user groups” to advise ongoing program changes (e.g., Harrison & Brandling., 2009); participant‐involved outreach strategies (e.g., Klemm et al., 2013); the inclusion of participants in program design and development (e.g., Bogart & Uyeda, 2009; Dworski‐Riggs & Langhout, 2010; Ward & Bailey, 2013; Weeks et al., 2010), program delivery (e.g., Baptiste et al., 2006; English et al., 2008; Noel, Rost, & Gromer, 2013), or program evaluation and communication (e.g., Checkoway & Richards‐Schuster, 2003; Shriberg et al., 2012; Trauth‐Nare & Buck, 2011); and engaging service providers in program implementation (e.g., Kegeles et al., 2012; L'Etang & Theron, 2012; Morisky et al., 2010; Rhodes et al., 2012). For example, in their discussion of the youth‐focused Lifting New Voices project, Checkoway and Richards‐Schuster (2003) explained that “each [participating] organization…formulated a plan, formed a steering committee, hired a youth organizer, and established a structure for implementation” (p. 28). The project “is evaluated by a participatory community‐based process which involves youth and adults in documenting their activities, assessing their experiences…” (p. 28). We also uncovered examples of arts‐based approaches in collaborative implementation, as in the case of DeMarco and Lanier's (2014) description of a study involving the use of a “film [to] help other women ﬁnd and use their voices to help decrease health disparities uniquely experienced by black women and perhaps others” (p. 117). Alternatively, Stewart, Riecken, Scott, Tanaka, and Riecken (2008) explain that artistic educational videos were created to improve health literacy and address health concerns among Indigenous youth, and others describe the use of photovoice in community and program assessment (e.g., Brazg et al., 2011; Dean, 2007; Guerra, Rodrigues, Demain, Figueiredo, & Sousa, 2013).

The benefits of collaborative implementation are noted by authors such as Ward and Bailey (2013), who point out that, “significantly women [prisoners] were clearly able to articulate strategies that helped them manage their self‐harm and were able to identify services they would access if available” (p. 313). Swaans, Broerse, Meincke, Mudhara and Bunders (2009) described extensive collaborative implementation efforts in their study of an agricultural intervention focused on food security for people living with HIV/AIDS. They also noted challenges with this approach, including persistent difficulties in engaging with the most stigmatized community members.

#### 3.2.5 Participatory dissemination

A key goal for most researchers is to disseminate findings in an impactful way. However, only 19% of studies we reviewed included discussions about participatory approaches to dissemination. The most common research focus where participatory approaches to dissemination were noted was in research related to child/adolescent development (28% of child/adolescent studies reviewed). Still, some researchers offered interesting examples of working with participants to disseminate findings and mobilize others into action. For example, youth researchers supported youth participants in: lobbying municipal agencies (e.g., boards of education; Lind, 2007; Maglajlic, 2010); disseminating their findings through animated films presented to local services and used in educational settings (Taggart et al., 2013); and developing project websites (Flicker et al., 2008). Importantly, in the last example, to compete with the marginalization that these youth often felt, and to communicate the value of their input, they were provided with honoraria for participation. Participants in other studies (e.g., Mordock & Krasny, 2001; Ross, 2011) have advocated for passing legislation for particular causes. Ross (2011), on remarking about the impact of youth involvement in a tobacco initiative, reported, “one of the coalition adults commented that she had never seen a Senator or representative agree so quickly to act for a community group and was convinced that the combination of the youth's energy and quality of their research inspired the senator to act on their behalf” (p. 694). In other cases, participants were involved in prioritizing and fostering identified outreach strategies (Foster‐Fishman et al., 2005; Klemm et al., 2013) and targets (Novek et al., 2012), or in determining strategies for communicating (Ball & Janyst, 2008; Flicker et al., 2008) and implementing (Bland & Atweh, 2007) findings. Teti et al. (2013) described HIV/AIDS participants’ involvement in a public art show as a form of disseminating findings. As one example, in a study on the effects of mining with the Mam Mayan Indigenous community in Guatemala, Caxaj et al. (2014) described working “in partnership with participants to plan knowledge translation activities that showcased the ﬁndings from the participants’ viewpoint in a way that was considered relevant and useful to the community” (p. 826). While participatory dissemination was not extremely common, the examples provided by researchers were diverse and compelling.

### 3.3 Exemplars of the application of participatory research principles in psychology

Our second research question was *Which studies can be considered exemplars in the application of PR principles across the six research areas of focus?* We included studies that met all three criteria for being exemplary, including: Rigorous design; community/participant involvement in all stages; and evidence that the research encourages trust, promotes reciprocal capacity building, and acknowledges power dynamics. We identified three exemplars that are indicative of the types of successes that can emerge from consistent and in‐depth collaboration with community resources and researchers. The exemplars substantiate the finding that when PR approaches are employed from the outset until the dissemination phase, meaningful and sustainable impacts are possible.

An article reporting on the participatory creation of a communications strategy targeting youth HIV/AIDS prevention (Maglajlić & RTK PAR UNICEF BiH Team, 2004) is one example that meets all three criteria for exemplary PR. Collaboration at all research stages was described (pp. 128–135), including a description of the responsibilities undertaken by various team members. Among the efforts to ensure rigor, Maglajlić and RTK PAR UNICEF BiH Team (2004) employed multiple methods (p. 133) and undertook extensive training with participant researchers (p. 129), a commitment that also contributed to meeting the criterion of “promoting reciprocal capacity building.” In particular, the structure of the project team included a head researcher with extensive PR experience, and three research site teams, each of which involved five young people nominated by relevant partner organizations to serve as team leads (who were compensated financially) and a volunteer group of 15–20 young people to be involved in the project as team members. The team leads attended two workshops on PR skills, and each site team met nine times over 6 months, with time consistently set aside to reflect on the process. Team members share in various roles (e.g., group facilitator, record keeper, and “devil's advocate”) throughout the 6 months. Together, the teams “decided what to research, how to research it, with whom and when, conducted the research and other activities with other adolescents in their town, made sense of the data and worked with the research team to develop the proposal for a prevention strategy” (p. 130). Overall, data collection methods involved focus group discussions, questionnaire surveys conducted at times and places (e.g., coffee shops, pool, and concerts) typically frequented by select adolescent populations, qualitative surveys, and “comment walls” (i.e., where participants shared their opinions on different topics). Participant quotes included in the article speak to the third criteria, where trust building is explicitly noted (p. 131). In the end, the research teams focused on various themes (i.e., sexually transmitted infections, human rights, HIV/AIDS, and substance misuse), aggregated the findings of their research, and used these findings to develop proposals to engage in peer education and media communication.

In the second example, Ball and Janyst (2008) describe deep collaboration between university and community partners across two research projects with Indigenous Peoples; one focused on the wellbeing of Indigenous children, and the other focused on Indigenous fathers’ engagement. The rigor in the studies reported in the article (criterion 1) is highlighted by the use of mixed and culturally relevant methods (p. 43), including face‐to‐face, semi‐structured interviews by volunteer community members; and documentation of existing resources within the communities. All procedures considered a traditionally collectivist worldview by focusing on families and communities as units of analysis and a history of traumatic experiences by avoiding triggering questions. In both projects, community benefit as a result of the research was prioritized, which speaks not only to participant involvement across stages of the research (criterion 2), but also to relationship building including by promoting trust and reciprocal capacity building (criterion 3). Ongoing consultations with community representatives ensured respectful data collection, analysis, interpretation, and knowledge mobilization. In the third example, Allen et al. (2014a), reporting on the “People Awakening Project”^6^, note: “[the] story of our work is thus that of an extended research relationship rather than a discrete intervention. Through this collaborative research relationship, we were able to generate a robust, locally developed theory of protective factors from suicide and alcohol abuse, and a culturally relevant evaluation of a strengths‐based, multi‐level, and community‐based preventive intervention” (p. 110). This study offered extensive details related to its rigor (criterion 1), persistent collaboration (criterion 2), and commitment to relationship building (criterion 3). It is worth noting that the ethical demands of contemporary research collaborations with Indigenous Peoples and Nations align fairly well with the criteria for exemplary PR. There are thus surprisingly few exemplars within our sample.

### 3.4 Limitations

A limitation of this review is that our approach to coding necessitated that authors offer descriptions or explanations of how they undertook or applied PR principles in the research. In other words, it was not sufficient to simply state that PR principles were employed. As such, an article was tagged, “no PR in study” if the authors claimed to use a participatory approach, but then offered no description of these efforts. Given the word limits in academic journals, it is possible that authors of such articles faced word constraints that prevented them from explaining their PR approaches in detail. Nevertheless, because the goals of this paper include better understanding and encouraging the application of PR approaches in psychology, our decision to include only studies that described participatory efforts was appropriate. A limitation of our decision to use a scoping (not systematic) review is that we have focused on providing a sufficient rather than exhaustive rationale for PR amongst psychology researchers. In other words, there are assuredly good PR examples not covered by this review. Finally, because of the time required to sort, analyze, and present the results, new studies following participatory principles may have emerged in the past 18 months. Nevertheless, we are confident that our results provide a current overview of the state of PR across the discipline of psychology.

## 4 DISCUSSION AND CONCLUSIONS

Our analysis framework and findings offer guidance for researchers and student trainees in psychology programs interested in pursuing more participatory approaches in their research. For undertaking meaningful PR, commitments to full inclusion of participants and reciprocity of benefit are essential. The findings highlight ways that researchers are applying PR principles in a wide range of psychology research. In general, exemplars include ongoing, culturally sensitive consultations with community members, multiple methods of data collection (e.g., focus groups, surveys, and semi‐structured interviews) within relevant communities, and community‐driven approaches throughout the research process. Based on our analysis, three areas, aligned with the goals laid out in the introduction of this paper, merit further attention. These are: complements and conflicts; fidelity to PR principles; and definitions and descriptions of “partners.”

### 4.1 Complements and conflicts

Across the six research areas of focus (see Table 1), we found examples of deep commitments to the benefits and ethics of PR. There was evidence of meaningful participatory commitments being taken up in all stages of research, from research design through the dissemination of findings. As noted above, researchers attributed a number of positive outcomes to their participatory approaches, including identifying appropriate research priorities, better understanding participants’ experiences, and complementing other research design and methodological approaches. Further, as discussed below, participants’ experiences – when understood as providing expertise – made important contributions to knowledge, which can otherwise be lost.

This is not to say that participatory approaches to research are without challenges. Indeed, this is a plausible part of the reason that many PR studies we reviewed are missing dimensions of participation at key stages in the research process, such as research design, data analysis, and dissemination of information. Key challenges included contending with persistent power imbalances between community and university‐based researchers, and seeking and maintaining consent from participants who are extremely marginalized. A commonly discussed approach to ameliorating these and other challenges was to engage an advisory group that could offer guidance throughout the research process. Furthermore, there were notable attempts to avoid tokenism by financially supporting community researchers, making sure to include a sufficient number of them (relative to university‐based researchers), and ensuring their authority early in a project, specifically on project design.

### 4.2 Fidelity to participatory research principles

Across the research areas of focus, just over 50% of articles located using the terms “participatory” and “research” were retained after our review of both the abstracts and methods sections of studies. As outlined in great detail in the introductory and methodological sections of this article, there are well‐established – if not universal – guidelines and principles for informing participatory researchers, including the importance of rigorous study design, full participant inclusion across all stages of the research (Viswanathan et al., 2004), and commitments to reciprocity and reciprocal capacity building (Belone et al., 2016). Most excluded studies were removed because they asserted that they had adhered to PR principles but offered no corresponding evidence or explanation. This suggests three possibilities. One is that researchers are – for reasons of space or otherwise – simply not describing their participatory efforts. To guard against this possibility in the future, we encourage psychologists across research areas to be more explicit about their use of participatory approaches. Doing so could provide more explicit guidance to those interested in pursuing PR, and more importantly, could ensure that the benefits of PR are being realized. A second possibility is that authors are using the terminology of PR inappropriately. For example, some studies described their methods as participatory but held focus groups designed by the university‐based researcher(s). We suggest that research participants’ involvement as subjects in qualitative data collection methods such as focus groups is not evidence of PR. Similarly, recruiting participants through a community agency may highlight the importance of a partner's engagement for research success, but is not in itself evidence of the application of PR principles. Related, it could also be the case that researchers are using the language of PR without actually undertaking the required work, a situation that may result from limited commitment to navigating the challenges that often accompany PR.

A third explanation for the disconnect between claims of PR and associated descriptions of such work is that authors may have intended to use participatory methods but were not successful in doing so because they were not able to foresee or overcome challenges presented by PR approaches. For example, it can be difficult to retain participants’ enthusiasm throughout a long research process (Levac, 2013; Torronen & Vornanen, 2014), and to allow for the circuitous path that PR takes (McIntyre, 2006), given the variable time and output pressures facing universities, communities, and participants. Nevertheless, reflecting on the process with participants, however difficult, is crucial and allows for growth as well as novel interactions between researchers, service and policy professionals, community members, and participants (Ozer & Wright, 2012). It may also be the case that graduate students and researchers in psychology have limited access to training in PR. This could be overcome with ongoing training through didactic lectures; role playing; group discussions on such topics as research fundamentals, research ethics, and participant recruitment; and discussions about specific protocols of particular studies (Livingston et al., 2014). Our findings provide some strategies and examples for addressing such challenges.

### 4.3 Definitions and descriptions of “partners”

Finally, our review highlights that while research “participants” in PR projects share the characteristic of being based in places other than postsecondary institutions, their characteristics are otherwise highly variable. For example, participants can be community organizations that provide services (e.g., Bowden et al., 2006; Checkoway & Richards‐Schuster, 2003), or service users themselves (e.g., Cotterell, 2008; Taggart et al., 2013). In research focused on youth/adolescent development, “participants” were sometimes youth (e.g., Flicker et al., 2008; Levac, 2013), but commonly parents, teachers, or youth‐serving organizations. Although it is difficult to offer universal guidance on establishing appropriate partnerships, a range of points merit attention. First, participatory researchers should remember that people with important experiential knowledge and expertise may not be affiliated with institutions and organizations. As well, organizational staff members with high levels of integrity may not fully understand the experiences of particular groups that they serve, including, for example, people living with HIV/AIDs, young people, or people who are transgender. Second, it is also the case that community organizations hold important knowledge and expertise and can thus be vital and reciprocal partners in research. A community's uptake of research may be more plausible through collaboration with an organization that is able to implement results. Third, participatory researchers may benefit from being aware of discussions about research insider/outsider positioning, but particularly the extent to which these boundaries are often fluid (Eppley, 2006), and how this fluidity can intersect with participatory research approaches (Spencer, 2015). These considerations, which may sit in tension with each other, lead to the conclusion that who counts as appropriate “participants” in PR requires careful consideration, and depends critically on the questions being asked and the purposes of the research.

Related to this point is the fact that “participants” across the studies we reviewed were described variously as co‐researchers, research team members, community researchers, peer researchers, and participants. While our emphasis in this study was not to review terminology, it is nevertheless important to point this out, if only to highlight the value of university‐based researchers being specific and descriptive about their language choices. In the future, it would be useful to try and understand the extent to which different terminology reflects varying levels of commitment to, or depth of, the PR process.

### 4.4 Ways forward

This article provides an overview – and a host of examples – of how participatory approaches to research are being employed across all stages of the research process in six research areas of focus in psychology research. The examples referenced throughout this manuscript demonstrate that research rigor can be maintained while fully involving partners across research stages. However, despite the range of interesting examples uncovered, there is relatively little evidence of the use of PR approaches, despite the ethical and empirical imperatives for its uptake. A key goal of this article is to offer ideas to researchers interested in advancing their capacity for, and practice of, participatory research. One possibility, to which we allude above, is better PR training for graduate students. Inviting research trainees to learn about participatory approaches can allow them to experience the benefits and learn to navigate the challenges. Having said that, the often‐longer timeframes for participatory research presents a barrier for students who are under intense time pressures to complete degrees for financial and regulatory reasons. As such, besides committing to student training, postsecondary institutions need to recognize and work to reduce related barriers to uptake. Another possibility is for researchers to encourage their universities to adjust reward structures that privilege output volume over impact. As Gelmon, Jordan, and Seifer (2013) explain, “institutions must adopt policies that value and reward [PR] as a form of scholarship…[and] provide structural supports for faculty who are developing [associated research] relationships…” (p. 64). A third suggestion is for postsecondary institutions to permit alternative formats for student theses and dissertations. Allowing students to produce research results in formats useful to community organizations and citizens will inevitably have the effect of facilitating broader research outreach. To advance PR uptake further still, we suggest that a (virtual) venue or forum dedicated to researchers interested in discussing their participatory approaches and challenges, and building meaningful research collaborations, could provide important training and peer mentorship and prove valuable for training students. Some national networks, such as Community‐Based Research Canada (CBRC) and Community Campus Partnerships for Health (CCPH) in the United States, could serve as useful starting points in this regard. We also acknowledge Lykes’ (2017) call to return to the activist roots of PR and suggest that while the challenges of isolating research impact are noteworthy, a useful subsequent scoping review could focus on the ways in which participatory approaches to research in psychology are bringing about meaningful change for participant researchers and their communities. Overall, the research presented in this article highlights both successes and promises of participatory approaches in psychology. Hopefully this encourages researchers to consider expanding their theoretical orientations and methodological toolkits.

## Footnotes

1 Cargo & Mercer (2008) identify 14 types of participatory research used in public health and suggest that all share similar interests and commitments. Besides participatory action research (Kemmis et al., 2000), other variants include feminist participatory action research (Frisby et al., 2009) and community‐based participatory research (Israel et al., 1998).

2 We are not aware of such studies, as of January 2019.

3 Often, barriers to full participation are imposed by university‐based researchers and their institutions. We discuss some of these barriers in the discussion section. However, it can also be the case that full PR is undesirable for participant researchers. For instance, in some cases, community members are not best positioned to undertake all stages of research due to competing priorities. For instance, as noted in a research report on the experiences of inner‐city residents in Winnipeg, Canada, “it takes all day to be poor” (Canadian Centre for Policy Alternatives Manitoba Office, 2009). This and other socioeconomic and political realities can impact the possibilities for participation. As well, participants may have different preferences for participation based on their age (Powers & Tiffany, 2006) or may be identified based on labels (e.g., consumer, service user, and patient) that impede genuine power sharing. Ultimately, it is community participants who should determine their participation preferences.

4 We recognize that describing research with Indigenous Peoples as an area of research focus could be interpreted as objectifying. However, our decision to examine research with Indigenous Peoples in this review of participatory research efforts in psychology is motivated explicitly by our strong assertion that research *on* Indigenous Peoples – rather than *with* them – is deeply problematic.

5 Throughout the findings section, references included in relation to various participatory principles and practices are not exhaustive. In other words, included references offer a sample of studies where the principle or practice being discussed can be found.

6 Five other articles in the same issue of the Journal of Community Psychology (September 2014, volume 54, issue 1–2) report on elements of the People Awakening Project, making it a useful and comprehensive overview of several aspects of participatory research.
